# Pharmacokinetic Interaction Between the MEK1/MEK2 Inhibitor Trametinib and Oral Contraceptives Containing Norethindrone and Ethinyl Estradiol in Female Patients With Solid Tumors

**DOI:** 10.1002/cpdd.1052

**Published:** 2022-02-14

**Authors:** Hendrik‐Tobias Arkenau, Donatienne Taylor, Xiaoying Xu, Shripad Chitnis, Casilda Llacer‐Perez, Kathleen Moore, Prasanna Kumar Nidamarthy, Palanichamy Ilankumaran, Judith De Vos‐Geelen

**Affiliations:** ^1^ Sarah Cannon Research Institute London UK; ^2^ Cancer Institute University College London London UK; ^3^ CHU UCL Namur, Site Sainte‐Elisabeth Namur Belgium; ^4^ Novartis Pharmaceuticals Corporation East Hanover New Jersey USA; ^5^ Novartis Institutes for BioMedical Research Cambridge Massachusetts USA; ^6^ Hospital Clinico Universitario Virgen de la Victoria Malaga Spain; ^7^ Stephenson Cancer Centre University of Oklahoma Oklahoma City Oklahoma USA; ^8^ Sarah Cannon Research Institute Nashville Tennessee USA; ^9^ Novartis Healthcare Pvt. Ltd. Hyderabad India; ^10^ Department of Internal Medicine Division of Medical Oncology GROW, School for Oncology and Developmental Biology Maastricht UMC+ Maastricht The Netherlands

**Keywords:** anaplastic thyroid cancer, MEK inhibitor, melanoma, non–small‐cell lung cancer, oral contraception, trametinib

## Abstract

This phase 1 postapproval study assessed the effect of the mitogen‐activated protein kinase kinase enzyme 1/enzyme 2 inhibitor trametinib (2 mg once daily, repeat dosing) on the pharmacokinetics of combined oral contraceptives (COCs) containing norethindrone (NE; 1 mg daily) and ethinyl estradiol (EE; 0.035 mg daily) in 19 female patients with solid tumors. Compared with NE/EE administered without trametinib, NE/EE administered with steady‐state trametinib was associated with a clinically nonrelevant 20% increase in NE exposure (area under the curve [AUC]) and no effect on EE exposure (geometric mean ratio [geo‐mean] of NE/EE + trametinib to NE/EE [90%CI]: NE AUC calculated to the end of a dosing interval at steady‐state [AUC_tau_] 1.20 [1.02‐1.41]; NE AUC from time zero to the last measurable concentration sampling time [AUC_last_] 1.2 [0.999‐1.45]; EE AUC_tau_ 1.06 [0.923‐1.22]; EE AUC_last_ 1.05 [0.883‐1.25]). Maximum serum concentration (C_max_) of NE increased by 13% and C_max_ of EE decreased by 8.5% when dosed with steady‐state trametinib compared with COCs administered alone (geo‐mean ratio [90%CI]: NE C_max_ 1.13 [0.933‐1.36]; EE C_max_ 0.915 [0.803‐1.04]). These results indicate that repeat‐dose trametinib does not lower exposure to NE or EE and, hence, is unlikely to impact the contraceptive efficacy of COCs. The pharmacokinetic parameters of trametinib and its metabolite M5 were consistent with historic data of trametinib alone. Coadministration of trametinib and COCs was generally well tolerated in this study, with observed safety signals consistent with the known safety profile of trametinib and no new reported safety events. Overall, the findings indicate that hormonal COCs can be coadministered in female patients who receive trametinib monotherapy without compromising the contraceptive efficacy.

Trametinib (Mekinist) is an oral, selective inhibitor of mitogen‐activated protein kinase kinase enzyme 1 (MEK1) and enzyme 2 (MEK2), which are components of the rat sarcoma virus (RAS)/rapidly accelerated fibrosarcoma (RAF)/mitogen‑activated protein kinase kinase (MEK)/extracellular signal‑regulated kinase (ERK) signaling pathway.^1^ Dysregulation of this pathway occurs in more than one‐third of all malignancies, and results from a variety of different genetic alterations including *BRAF^V600^* mutations.^2^, ^3^ Inhibition of MEK is therefore an attractive strategy for targeted treatment of tumors harboring mutations in the RAS/RAF/MEK/ERK pathway.

Trametinib has demonstrated clinical activity as single‐agent in patients with *BRAF* ^V600E/K^‐mutated unresectable or metastatic melanoma,^4^, ^5^ as well as in combination with the BRAF inhibitor dabrafenib in patients with unresectable or metastatic, *BRAF* ^V600^‐mutated melanoma, either as first‐line treatment of BRAF inhibitor–naive patients^6^, ^7^, ^8^, ^9^ or as adjuvant therapy,^10^ in patients with metastatic *BRAF* ^V600E^‐mutated non–small‐cell lung cancer,^11^, ^12^, ^13^ and in patients with locally advanced or metastatic *BRAF* ^V600E^‐mutated anaplastic thyroid cancer.^14^

The pharmacokinetics (PK) of trametinib in humans have been established in a phase 1 study in patients with advanced solid tumors, which showed that at the recommended phase 2 dose (2 mg once daily), the mean peak plasma concentration (C_max_) of trametinib is 22.2 ng/mL, with a time to reach C_max_ (t_max_) of 1.5 hours, a half‐life (t_1/2_) of 5.3 days, and a flat exposure profile and small peak:trough ratio (1.8).^15^, ^16^ A human metabolism and disposition study showed that trametinib is metabolized by non–cytochrome‐mediated mechanisms, mainly involving deacetylation via hydrolytic enzymes alone or in combination with glucuronidation.^17^ The major component in plasma is unchanged trametinib, accounting for 26% to 72% of drug‐related material. The pharmacologically active metabolite M5 (formerly called M1), formed through deacetylation and accounting for <11% of drug‐related material in plasma, demonstrated approximately equally potent activity in vitro as the parent (half maximal inhibitory concentration to inhibit phospho‐MEK1, trametinib 7.0 ± 0.1 nM; M5 9.0 ± 1.0 nM).^17^

Based on in vitro studies, trametinib has been shown to be a low‐level inducer of cytochrome P450 (CYP) 3A4 and an inhibitor of the UDG‐glucosyltransferase UGT1A1, with no inhibitory activity of CYP1A2, CYP2A6, CYP2B6, CYP2D6, or CYP3A4.^18^, ^19^ In vitro studies in hepatocytes established the half maximal effective concentration (EC_50_) of trametinib for CYP3A4 induction as 1.7 μM, with a maximal effect of 69% of that of the strong CYP3A4 inducer rifampicin (Novartis, unpublished data). However, the clinically relevant systemic exposure for therapeutic activity of trametinib is ≈0.04 μM (based on the C_max_ of trametinib of 22.2 ng/mL^15^), which is lower than the observed in vitro CYP3A4 induction value. Therefore, based on its clinically relevant systemic exposure and its EC_50_ in vitro, trametinib is not expected to be an inducer of CYP3A4 in vivo.

Female patients of reproductive potential receiving trametinib are advised to use effective contraception, since maternal and developmental toxicities have been observed in animal reproduction studies of trametinib (Novartis, unpublished data). Combined oral contraceptives (COCs) containing norethindrone (NE) and ethinyl estradiol (EE) are widely used in women of childbearing potential. Both NE and EE are substrates of CYP3A4 and undergo oxidative and reductive metabolism.^20^ Because a potential effect of trametinib on the metabolism of NE and EE might lower their systemic exposure, which could lead to contraception failure, a drug‐drug interaction (DDI) between trametinib and NE or EE in vivo needs to be ruled out. Furthermore, as there are no clinical studies of trametinib in women of childbearing potential, additional evidence on the PK and safety of trametinib when coadministered with COCs is important to inform the use of trametinib in female patients who take COCs.

The main objective of this study was to assess the effect of repeat‐dose trametinib (2 mg once daily) on the PK of COC (NE + EE) in female patients with solid tumors. In addition, the study also evaluated the PK of trametinib and its metabolite M5 when used in combination with COCs, as well as the safety and tolerability of trametinib in female patients with solid tumors taking COCs.

## Methods

### Ethics

The study was conducted in the United Kingdom (Sarah Cannon Research, UK, London), The Netherlands (Maastricht UMC+, Limburg), Belgium (CHU UCL Namur site Sainte‐Elisabeth, Namur), Spain (Hospital Clinico Universitario Virgen de la Victoria, Malaga), and the United States (Stephenson Cancer Center, Oklahoma City, Oklahoma). The protocol (NCT02705963) for this study was reviewed and approved by an independent ethics committee at each site (Bristol Research Ethics Committee Centre, Bristol, United Kingdom; Stichting Beoordeling Ethiek Biomedish Onderzoek, Assen, The Netherlands; Comité d'Ethique du CHU UCL Namur site Sainte Elisabeth, Namur, Belgium; CEIm Provincial de Málaga Unidad, Hospital Regional Universitario de Málaga Ethics Committee for Research with Medicines, Malaga, Spain; and IntegReview IRB, Austin, Texas) before initiation of the trial. All enrolled patients provided written informed consent. The study was conducted in compliance with the principles of the Declaration of Helsinki, using Good Clinical Practice according to the International Council for Harmonization Tripartite Guidelines.

### Study Design

**Figure 1:**
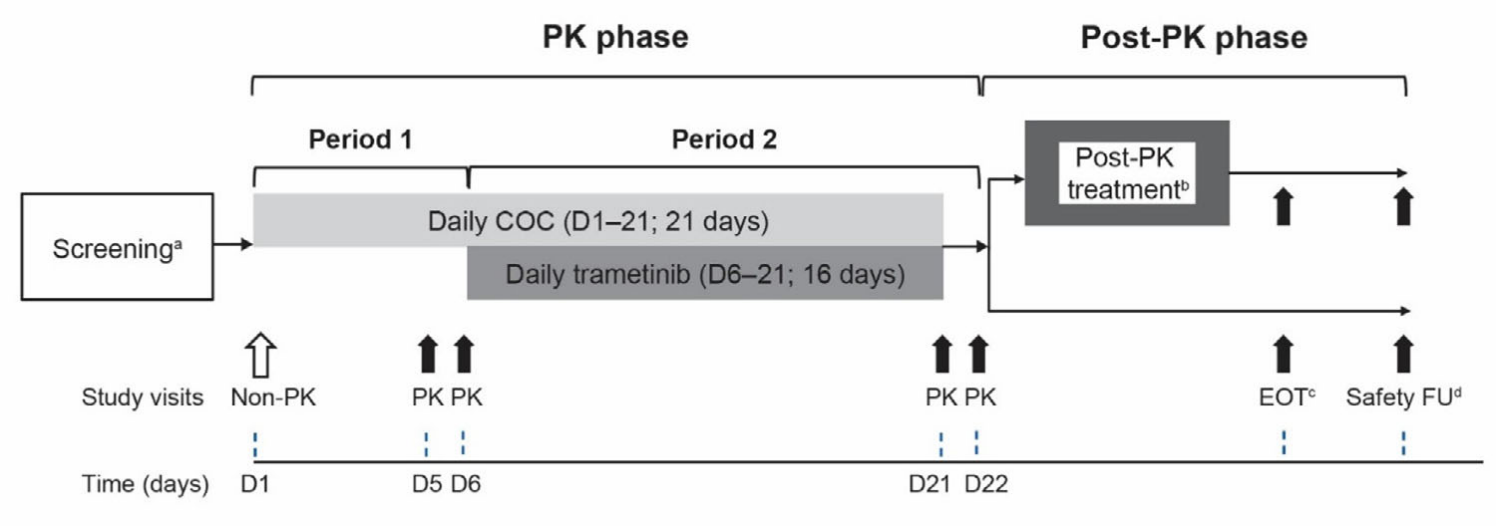
Study design. COC consisted of 1 mg of norethindrone and 0.035 mg of ethinyl estradiol daily; trametinib dosing was 2 mg daily. ^a^Within 30 days of day 1. ^b^After completion of the PK phase, patients could continue trametinib if they derived benefit from it, unless they experienced disease progression or intolerance of study drug, withdrew consent, or were lost to follow‐up as per the investigator's discretion; continuation of COC after PK phase was optional. During the post‐PK phase, visits were every 4 weeks. ^c^Within 7 days after last dose of study treatment. ^d^30 days after last dose of study treatment (follow‐up on adverse events, serious adverse events, and concomitant medications). COC indicates combined oral contraceptives; D, day; EOT, end of treatment; FU, follow‐up; PK, pharmacokinetics.

This was a phase 1, open‐label, single‐sequence, 2‐period crossover, multicenter study evaluating the effect of repeat‐dose trametinib on the steady‐state PK of NE/EE in female patients with solid tumors. The study consisted of a PK phase involving treatment period 1 (days 1‐5) and treatment period 2 (days 6‐22), followed by a post‐PK phase during which patients could continue trametinib until disease progression, intolerance of study drug, withdrawal of consent, or lost to follow‐up as per the investigator's discretion (Figure 1). An end‐of‐treatment visit was conducted within 7 days of the last dose of the study treatment, and a final safety follow‐up was conducted 30 days after the last dose of study treatment. The single‐sequence, 2‐period study design was chosen due to the long t_1/2_ of trametinib (5.3 days), which ruled out a 2‐way crossover design as this would have required a long, ethically unacceptable washout period in patients with cancer. The 2‐period PK phase was designed to allow assessment of EE and NE steady‐state PK when used in the absence of trametinib (steady state of NE and EE achieved at ≈days 5‐6, based on the mean t_1/2_ of 20 hours for EE and 8.5‐11 hours for NE), and when used in combination with trametinib (trametinib steady state achieved at approximately day 15). The PK of trametinib and M5 were assessed during dosing with COC.

### Patients

Participants were required to be female, aged ≥18 years and <59 years, postmenopausal or of childbearing potential, and with a confirmed diagnosis of a solid tumor that is refractory to standard therapies or for which there is no approved therapy. Patients also had to be on a stable COC regimen of 1 mg of NE and 0.035 mg of EE (1/35 NE/EE), willing to switch from a stable regimen of an alternate oral contraceptive to a COC regimen of 1/35 NE/EE, or willing to start a COC regimen of 1/35 NE/EE. Patients were required to have no prior treatment‐related toxicities of grade >1 (except alopecia) at the time of enrollment and have Eastern Cooperative Oncology Group performance status 0 to 1. Key exclusion criteria were prior exposure to an MEK inhibitor; *BRAF^V600E^*‐mutant melanoma that had failed prior BRAF inhibitor therapy, metastatic pancreatic cancer (due to minimal clinical activity of trametinib in these patients); known or suspected carcinoma for which administration of a COC would be contraindicated (ie, breast cancer or other estrogen‐dependent carcinoma and hepatocellular carcinoma); a history of any conditions where administration of a COC would be contraindicated; or participation in a clinical study and having received investigational drug(s) within 30 days, 5 half‐lives, or twice the duration of the biological effect of trametinib, whichever was longer, before the first dose of the study treatment in this study.

### Treatments and Dosing

Treatments were administered in a sequential order, with COC dosing (1 mg NE and 0.035 mg EE daily) initiated on day 1 and trametinib dosing (2 mg daily) initiated on day 6. Both regimens were continued until day 21 (Figure 1). Treatment dosing was based on the approved dose of trametinib for patients with metastatic melanoma (2 mg daily) and the approved and commonly prescribed dose of COCs (1 mg NE and 0.035 mg EE daily). Trametinib dose reductions were permitted in patients who could not tolerate the protocol‐specified dosing schedule. Patients on 1.5 mg of trametinib remained evaluable for the PK phase of the study. Treatments were self‐administered at home, except for days 1, 5, 6, 21, and 22 when treatments were administered at the Clinical Research Unit. Concomitant treatment with other anticancer agents (eg, chemotherapy, immunotherapy, biologic therapy, and/or hormone therapy) was not allowed, but patients received full supportive care throughout the study.

### Study End Points

The study primary end point was assessment of the primary PK parameters AUC_tau_, AUC_last_, C_max_, and t_max_ of NE and EE. Secondary and exploratory end points included steady‐state PK parameters of M5 and the safety of trametinib in patients who received trametinib in combination with COCs. Adverse events (AEs) of special interest included cardiac‐related events, bleeding events, hepatic events, hypersensitivity, hypertension, ocular events, pneumonitis, skin‐related toxicities, and venous thromboembolism.

### Pharmacokinetic Assessments

PK was assessed on days 5 and 6 (EE, NE) and on days 21 and 22 (EE, NE, trametinib, M5), with blood samples collected before dosing (days 5, 6, 21, 22), and at 0.5, 1, 1.5, 2, 2.5, 3, 4, 6, 8, and 10 hours after dosing (days 5 and 21). Plasma concentrations of NE, EE, trametinib, and M5 were measured using validated liquid chromatography–‐tandem mass spectrometry (LC‐MS/MS). Plasma sample analysis of NE and EE was based on a previously published bioanalytical method^21^ with minor modifications. NE and EE were extracted from human plasma by supported liquid extraction after the addition of isotopically labeled internal standards ([^13^C2]‐NE and EE‐d4). Extracts were analyzed using LC‐MS/MS. The analytical method was validated with an 8‐point calibration curve from 50 to 25 000 pg/mL for NE, and from 2.50 to 500 pg/mL for EE. The lower limit of quantitation (LLOQ) was 50.0 pg/mL for NE, and 2.50 pg/mL for EE using a 250 μL aliquot of human plasma. For each run, the accuracy of each sample was within the range of ±15.0% bias. For trametinib and M5 plasma sample analyses, trametinib, M5, and their internal standards ([^13^C6]–GSK1120212 and [^13^C6]–GSK1790627, respectively) were isolated from human plasma (containing dipotassium ethylenediaminetetraacetic acid as anticoagulant) by liquid/liquid extraction. After evaporation under nitrogen, the residue was reconstituted with 200 μL of aqueous acetonitrile (1:1 v/v). The reconstituted extract was injected into an LC‐MS/MS system (3500 V, 550°C; API4000, Sciex, Framingham, Massachusetts) using an ACQUITY UPLC BEH C18 column (50 × 2.1 mm, 1.7 μm particle size; Waters Corp., Milford, Massachusetts). Mobile phases contained water with 0.1% formic acid (mobile phase A), and acetonitrile with 0.1% formic acid (mobile phase B). The following mass spectrometry transitions were monitored: mass/charge ratio (m/z) 616–491 for trametinib and m/z 622–497 for its internal standard, and m/z 572 to 489 for M5 and m/z 578 to 495 for its internal standard. Results were calculated using peak area ratios of analyte to internal standard, and calibration curves were generated using a weighted (1/×2) linear least‐squares regression. Aliquots of blank human plasma from 6 different individuals were tested for endogenous interferences. In all cases, trametinib, M5, and internal standard regions were free from significant interference (<20.0% of the response from the single LLOQ used and <5.0% of internal standard response in the control 0 sample). The accuracy of each sample was within the range of ±15.0% bias. The LLOQ was 0.25 ng/mL for trametinib, and 0.05 ng/mL for M5. Linear calibration ranges were 0.25 to 250 ng/mL for trametinib, and 0.05 to 25.0 ng/mL for M5. The PK parameters were determined for all PK evaluable patients by noncompartmental methods using Phoenix WinNonlin (Pharsight, Mountain View, California) or other appropriate software.

### Statistical Analysis

Assuming a maximum intrasubject variability for NE and EE of 17%, and a true ratio of COC + trametinib to COC of 1, a sample size of ≈20 enrolled patients, resulting in a minimum of 12 patients completing the PK phase of the study (assumed dropout rate, 40%), was estimated to be sufficient to interpret the results with reasonable precision based on 90%CIs. The PK parameters were summarized using the geometric mean (geo‐mean), geometric coefficients of variation, median, minimum, and maximum. Baseline characteristics are presented as frequencies and percentages for categorical data, and as median, minimum, and maximum for continuous data. The full analysis set and the safety set included all patients who received at least 1 dose of any study treatment (trametinib or COC). The PK analyses of NE and EE (COC PK analysis set) included all patients who had an evaluable PK profile for at least 1 period, received the planned dose of COC for 5 consecutive days during period 1 (including day 5), received the planned dose of COC for at least 5 consecutive days during period 2 (including day 21), did not vomit within 4 hours after dosing of a COC on day 5 or day 21, and provided at least 1 primary PK parameter. PK analyses of trametinib and M5 (trametinib/M5 PK analysis set) included all patients who had an evaluable PK profile for at least 1 period, did not vomit within 4 hours after dosing of trametinib on day 21, and provided at least 1 primary PK parameter. Values below the LLOQ were treated as 0. Zero concentrations were considered as missing for calculations of the geometric means and geometric CV%. No formal statistical hypothesis testing for the effect of trametinib on NE/EE exposure was performed, and an estimation approach was adopted. For the primary PK parameters AUC_tau_, AUC_last_, and C_max_ of NE and EE, treatment differences were estimated using a linear mixed effects model. Trametinib + COC on day 21 was considered the test treatment and COC alone on day 5 as the reference treatment. The linear mixed‐effects model was fitted to the log‐transformed PK parameters of the COC, including treatment as a fixed effect and patient as a random effect. Point estimates of the treatment difference (test‐reference) and the corresponding 2‐sided 90%CI were calculated and antilogged to obtain the point estimate. For the primary PK parameter t_max_ of NE and EE, the median and range of the differences were calculated between the test and reference treatments. All analyses were performed using SAS version 9.4 (SAS Institute, Cary, North Carolina).

## Results

### Patient Disposition, Exposure, and Baseline Characteristics

Overall, 19 patients were enrolled in the study; of these, 14 (73.7%) patients completed the 2‐period PK phase (see also Figure 1). Five (26.3%) patients discontinued the study during the PK phase due to AEs (n = 3) or progressive disease (n = 2). A total of 16 patients entered the post‐PK phase (including patients who had not completed the PK phase); during the post‐PK phase, all 16 patients continued trametinib treatment, and no patient continued the COC. All 16 patients discontinued from the post‐PK phase due to progressive disease (n = 7), AEs (n = 3), study termination by the sponsor (n = 3), death (n = 1), physician decision (n = 1), or patient/guardian decision (n = 1). During period 1 of the PK phase, all 19 patients received at least 1 dose of NE/EE; of these, 18 patients received all planned doses of NE/EE for 5 consecutive days. The PK analysis set of period 1 (NE/EE) included 16/19 patients; 3 patients were not included because of study discontinuation during period 1 (n = 1), concentration data up to 1 hour after dosing only (n = 1), or protocol violation (received trametinib on day 5 instead of day 6; n = 1). During period 2 of the PK phase, 17 patients received trametinib as per protocol and 14 patients received both trametinib and the planned doses of NE/EE for 5 consecutive days. The PK analysis set of period 2 (NE/EE + trametinib) included 14/19 patients; 5 patients were excluded from this analysis as they did not have an evaluable PK profile for period 2. Overall, the median actual dose of trametinib was 2.0 mg/day (range, 1.4‐2.0 mg/day) and the median duration of exposure to trametinib was 6.1 weeks (range, 2‐22 weeks). At baseline, the median age was 49 years (range, 34‐59 years) and the most common cancer types were colon and rectum (21% each), mostly adenocarcinoma (63%) (Table 1).

**Table 1:** Summary of Baseline Demographics and Patient Characteristics (FAS)

	All patients (N = 19)
Age, y	
Median (range)	49 (34‐59)
Mean (SD)	47.3 (7.96)
18 to <65, n (%)	19 (100)
ECOG performance status, n (%)	
0	11 (58)
1	8 (42)
Primary site of cancer, n (%)	
Colon	4 (21)
Rectum	4 (21)
Cervix	3 (16)
Ovary	2 (11)
Others^a^	6 (32)
Histology/cytology, n (%)	
Adenocarcinoma	12 (63.2)
Neuroendocrine carcinoma	2 (10.5)
Adenosarcoma	1 (5.3)
Leiomyosarcoma	1 (5.3)
Serous adenocarcinoma	1 (5.3)
Other	2 (10.5)
Number of metastatic sites of cancer, median (range)	3 (1‐6)
Stage at study entry, n (%)	
Stage IIIC	1 (5)
Stage IV/IVB	18 (95)
Time since most recent relapse/progression to the first study treatment, median (range), months	2 (1‐6)
Prior antineoplastic therapy (received by >20% of patients)	
Any ATC medication	19 (100)
Surgery	15 (79)
Radiotherapy	6 (32)
Platinum compounds	18 (95)
Pyrimidine analogues	12 (63)
Monoclonal antibodies	9 (47)
Irinotecan	8 (42)
Taxanes	7 (37)
Detoxifying agents for antineoplastic treatment	6 (32)
Antineovascularization agents	5 (26)

ATC, anatomical therapeutic chemical; ECOG, Eastern Cooperative Oncology Group; FAS, full analysis set; SD, standard deviation.

Prior antineoplastic medications were coded using the World Health Organization Drug Reference List, which employs the ATC Classification System.

^a^ Includes adrenal cancer (n = 1), ampulla of Vater (n = 1), anorectal (n = 1), lung (n = 1), pancreas (n = 1), and uterus (n = 1).

### Effect of Trametinib on NE

**Figure 2:**
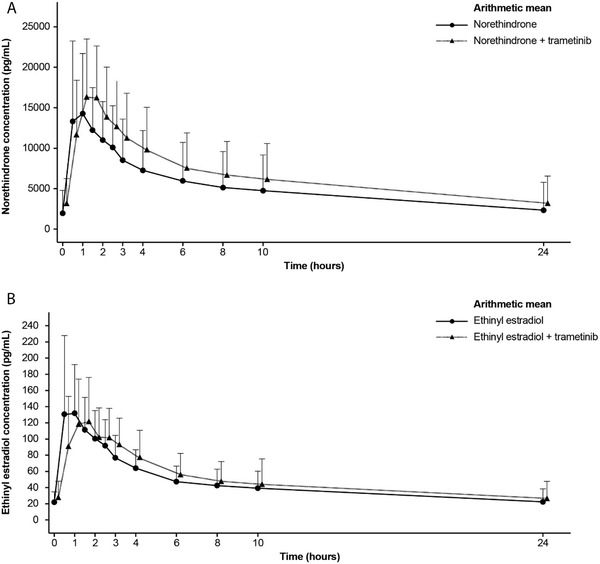
Plasma concentration–time profiles for norethindrone (A) and ethinyl estradiol (B) when administered with and without trametinib (pharmacokinetic analysis set–combined oral contraceptive). The arithmetic means with upper standard deviations are shown.

When NE/EE was coadministered with trametinib, the mean plasma concentration‐time profile of NE showed slightly higher C_max_ and AUC than when NE/EE was administered without trametinib (Figure 2a). Likewise, the geo‐mean of the primary NE PK parameters AUC_tau_, AUC_last_, and C_max_ were higher with NE/EE + trametinib than with NE/EE alone (Table 2). A statistical analysis showed that at steady‐state trametinib, NE exposure was 20% higher compared with NE/EE administered alone (geo‐mean ratio [90%CI] of NE/EE + trametinib to NE/EE: NE AUC_tau_ 1.20 [1.02‐1.41]; NE AUC_last_ 1.2 [0.999‐1.45]) (Table 3). Maximum serum concentration of NE increased by 13% in the presence of trametinib compared with NE/EE administered alone (geo‐mean ratio of NE/EE + trametinib to NE/EE: NE C_max_ 1.13 [0.933‐1.36]) (Table 3). Median t_max_, median t_1/2_, and geo‐mean total body clearance of drug from the plasma of NE were similar with and without trametinib, whereas geo‐mean AUC_inf_ and geo‐mean trough concentration of NE were higher in the presence of trametinib (Table 2), which is consistent with the observed increase in AUC_tau_. Therefore, when NE/EE was administered with trametinib, there was no reduction, but a 20% increase in NE exposure compared with NE/EE administered alone.

**Table 2:** Summary of PK Parameters of Norethindrone (NE) and Ethinyl Estradiol (EE) When NE/EE Is Administered Alone or With Trametinib (COC PK Analysis Set)

PK Parameters for NE	NE	NE + Trametinib
AUC_tau_, pg • h/mL		
n	16	14
Geo‐mean (geo‐CV%)	107 000 (77.6)	128 000 (67.0)
Mean (SD)	135 000 (102 000)	152 000 (96 200)
AUC_last_, pg • h/mL		
n	16	14
Geo‐mean (geo‐CV%)	103 000 (86.9)	123 000 (71.3)
Mean (SD)	134 000 (104 000)	149 000 (99 700)
C_max_, pg/mL		
n	16	14
Geo‐mean (geo‐CV%)	15 100 (50.7)	17 000 (36.1)
Mean (SD)	16 800 (7770)	18 000 (6340)
t_max_, h		
n	16	14
Median (range)	1.00 (0.50‐24)	1.02 (0.93‐2)
C_trough_, pg/mL		
n	16	14
Geo‐mean (geo‐CV%)	1060 (165.5)	1990 (140.5)
Mean (SD)	1940 (2850)	3040 (3110)
AUC_inf_, pg • h/mL		
n	8	6
Geo‐mean (geo‐CV%)	110 000 (79.1)	127 000 (80.2)
Mean (SD)	136 000 (96 500)	153 000 (95 200)
t_½_, h		
n	8	6
Median (range)	9.22 (4.31‐12.3)	9.06 (2.72‐11.3)
CL/F (L/h)		
n	9	6
Geo‐mean (geo‐CV%)	9.41 (76.9)	9.02 (72.6)

PK parameters for EE^a^	EE	EE + trametinib
AUC_tau_, pg • h/mL		
n	16	14
Geo‐mean (geo‐CV%)	1020 (59.5)	1050 (49.2)
Mean (SD)	1160 (567)	1170 (658)
AUC_last_, pg • h/mL		
n	16	14
Geo‐mean (geo‐CV%)	983 (70.0)	998 (53.1)
Mean (SD)	1150 (596)	1130 (679)
C_max_, pg/mL		
n	16	14
Geo‐mean (geo‐CV%)	135 (49.6)	120 (40.0)
Mean (SD)	151 (88.4)	129 (54.8)
t_max_, h		
n	16	14
Median (range)	0.98 (0.50‐2.50)	1.48 (0.52‐2.58)
C_trough_, pg/mL		
n	16	14
Geo‐mean (geo‐CV%)	21.7 (43.0)	21.7 (67.8)
Mean (SD)	22.2 (12.6)	26.2 (18.8)

AUC, area under the concentration‐time curve; AUC_last_, area under the concentration‐time curve from time 0 to the last measurable concentration sampling time; AUC_tau_, area under the concentration‐time curve calculated to the end of a dosing interval (tau) at steady state; CL/F, total body clearance of drug from the plasma; C_max_, maximum (peak) observed plasma concentration; COC, combined oral contraceptive; C_trough_, plasma concentration at time 0; EE, ethinyl estradiol; geo‐CV, geometric coefficient of variation; geo‐mean, geometric mean; n, number of patients with corresponding evaluable PK parameters; NE, norethindrone; PK, pharmacokinetics; SD, standard deviation; t_1/2_, elimination half‐life; t_max_, time to peak concentration.

For the PK analysis of period 1 (NE/EE), 3 of 19 patients were excluded due to lack of evaluable PK profile for period 1 (n = 2) or protocol violation (n = 1). For the PK analysis of period 2 (NE/EE + trametinib), 5 of 19 patients were excluded due to lack of evaluable PK profile for period 2.

^a^ The PK parameters AUC_inf_, CL/F, and t_1/2_ of EE could be determined in a limited number of patients only (n ≤ 3) in both periods because either extrapolated AUC was >20% or the regression coefficient was <0.75; these results are therefore not reported.

**Table 3:** Comparison of Norethindrone (NE) and Ethinyl Estradiol (EE) Primary PK Parameters When NE/EE Is Administered Alone or With Trametinib (COC PK Analysis Set)

Norethindrone (NE) PK Parameters						
				Treatment Comparison		
NE Parameter (Unit)	Treatment	n	Adjusted Geo‐Mean	Comparison	Geo‐Mean Ratio	90%CI
AUC_tau_, pg • h/mL	NE/EE	16	110 000	NE/EE + T vs NE/EE	1.20	1.02‐1.41
	NE/EE + T	14	131 000			
AUC_last_, pg • h/mL	NE/EE	16	106 000	NE/EE + T vs NE/EE	1.20	0.999‐1.45
	NE/EE + T	14	127 000			
C_max_, pg/mL	NE/EE	16	15 200	NE/EE + T vs NE/EE	1.13	0.933‐1.36
	NE/EE + T	14	17 100			

Ethinyl Estradiol (EE) PK Parameters						
				Treatment Comparison		
EE Parameter (Unit)	Treatment	n	Adjusted Geo‐Mean	Comparison	Geo‐Mean ratio	90%CI
AUC_tau_, pg • h/mL	NE/EE	16	1060	NE/EE + T vs NE/EE	1.06	0.923‐1.22
	NE/EE + T	14	1120			
AUC_last_, pg • h/mL	NE/EE	16	1020	NE/EE + T vs NE/EE	1.05	0.883‐1.25
	NE/EE + T	14	1070			
C_max_, pg/mL	NE/EE	16	140	NE/EE + T vs NE/EE	0.915	0.803‐1.04
	NE/EE + T	14	128			

AUC, area under the concentration‐time curve; AUC_last_, area under the concentration‐time curve from time 0 to the last measurable concentration sampling time; AUC_tau_, area under the concentration‐time curve calculated to the end of a dosing interval (tau) at steady state; CI, confidence interval; C_max_, maximum (peak) observed plasma concentration; COC, combined oral contraceptive; EE, ethinyl estradiol; geo‐mean, geometric mean; NE, norethindrone; PK, pharmacokinetic; T, trametinib.

Geo‐mean derived from model‐based analysis, including the respective PK parameter as a dependent variable, treatment as a fixed factor, and patient as a random factor.

n, number of observations used for the analysis.

### Effect of Trametinib on EE

The mean plasma concentration time profile (Figure 2b), as well as the primary EE PK parameters AUC_tau_, AUC_last_, C_max_, and t_max_ (Table 2) were similar with NE/EE + trametinib and NE/EE alone. A statistical analysis showed that at steady‐state trametinib, EE exposure was 5% to 6% higher compared with NE/EE administered alone (geo‐mean ratio [90%CI] of NE/EE + trametinib to NE/EE: EE AUC_tau_ 1.06 [0.923‐1.22]; EE AUC_last_ 1.05 [0.883‐1.25]) (Table 3). Maximum serum concentration of EE decreased by 8.5% in the presence of trametinib compared with NE/EE administered alone (geo‐mean ratio of NE/EE + trametinib to NE/EE: EE C_max_ 0.915 [90%CI, 0.803‐1.04]) (Table 3). Geo‐mean trough concentration of EE was similar between NE/EE with and without trametinib (Table 2). The EE PK parameters AUC_inf_, total body clearance of drug from the plasma, and t_1/2_ could only be determined in a limited number of patients for both periods, because either extrapolated AUC was >20% or the regression coefficient was <0.75; these results are therefore not reported.

### Effect of NE/EE on Trametinib and M5

The PK parameters of trametinib and its metabolite M5 when trametinib is coadministered with NE/EE are shown in Table 4. For trametinib, geo‐mean AUC_tau_ was 398  ng • h/mL, geo‐mean AUC_last_ was 346 ng • h/mL, geo‐mean C_max_ was 27.4 ng/mL, and median t_max_ was 2.01 hours. For M5, geo‐mean AUC_last_ was 51.5 ng h/mL, geo‐mean C_max_ was 3.75 ng/mL, and median t_max_ was 2.50 hours. The mean metabolite ratio (ratio of exposure of M5 to trametinib) was 0.20. Other PK parameters for trametinib and M5 could not be determined because either the AUC extrapolation was >20% or the regression coefficient was <0.75.

**Table 4:** Summary of PK Parameters of Trametinib and Its Metabolite M5 (Trametinib/M5 PK Analysis Set)

PK Parameter	Trametinib (n = 14)	M5 (n = 11)
AUC_tau_, ng • h/mL		
Geo‐mean (geo‐CV%)	398 (32.4)	NA
Mean (SD)	416 (123)	NA
AUC_last_, ng • h/mL		
Geo‐mean (geo‐CV%)	346 (51.5)	51.5 (100.7)
Mean (SD)	382 (162)	69.7 (56.0)
C_max_, ng/mL		
Geo‐mean (geo‐CV%)	27.4 (35.4)	3.75 (58.1)
Mean (SD)	28.9 (9.56)	4.29 (2.48)
t_max_, h		
Median (range)	2.01 (1.48‐3.00)	2.50 (1.55‐3.00)
C_trough_, ng/mL		
Geo‐mean (geo‐CV%)	13.1 (31.2)	ND^a^
Mean (SD)	13.7 (4.04)	ND^a^

AUC, area under the concentration‐time curve; AUC_last_, area under the concentration‐time curve from time 0 to the last measurable concentration sampling time; AUC_tau_, area under the concentration‐time curve calculated to the end of a dosing interval (tau) at steady‐state; C_max_, maximum (peak) observed plasma concentration; C_trough_, plasma concentration at time 0; geo‐CV, geometric coefficient of variation; geo‐mean, geometric mean; M5, metabolite of trametinib; n, number of subjects with corresponding evaluable PK parameters; NA, not applicable; ND, not determined; PK, pharmacokinetics; SD, standard deviation; t_max_, time to peak concentration.

Overall, 14 patients completed the PK phase and were analyzed for trametinib PK parameters; of these, 11 patients were analyzed for M5 PK parameters; 3 patients had their samples outside of the stability period and were excluded from the M5 analysis.

^a^ These PK parameters could be determined in a limited number of patients only because either the AUC extrapolation was >20% or the regression coefficient was <0.75; these results are therefore not reported.

### Safety

All 19 patients reported at least 1 AE during the PK phase and post‐PK phase (Table 5). The most common (reported in >20% of patients) any grade AEs were rash (n = 10; 52.6%), nausea (n = 8; 42.1%), diarrhea (n = 6; 31.6%), vomiting (n = 5; 26.3%), anemia (n = 4; 21.1%), increased blood alkaline phosphatase (n = 4; 21.1%), and stomatitis (n = 4; 21.1%). Overall, 14 patients had at least 1 grade ≥3 AE. The most common (reported in >10% of patients) grade ≥3 AEs were diarrhea, ascites, and hyponatremia, which were all reported in 2 patients (10.5%) each. Overall, 17 patients had at least 1 AE suspected to be related to the study drug (trametinib and/or COC), as assessed by the investigator. The most common (reported in ≥25% of patients) AEs considered to be study drug‐related were rash (n = 10; 52.6%), diarrhea (n = 5; 26.3%), and nausea (n = 5; 26.3%). Eight patients had at least 1 drug‐related grade ≥3 AE, including 2 patients (10.5%) with diarrhea, and 1 patient (5.3%) each with fatigue, aspartate aminotransferase (AST) increased, alanine aminotransferase (ALT) increased, blood pressure increased, hypoalbuminemia, hyponatremia, ileus, and myositis.

**Table 5:** Summary of Adverse Events (Safety Set)

	All Patients (N = 19)	
AE category, number of patients (%)	Any grade	Grade ≥3
AEs	19 (100)	14 (73.7)
Treatment related	17 (89.5)	8 (42.1)
SAEs	8 (42.1)	8 (42.1)
Treatment related	2 (10.5)	2 (10.5)
AEs leading to discontinuation	4^a^ (21.1)	
Treatment related	4^a^ (21.1)	
AEs leading to dose adjustment/interruption	10 (52.6)	

AE, adverse event; COC, combined oral contraceptive; PK, pharmacokinetic; SAE, serious adverse event.

Treatment‐related events could be related to trametinib and/or COC. Treatment discontinuations could involve discontinuation of trametinib and/or COC.

^a^ Includes 3 discontinuations of trametinib (all discontinued during post‐PK phase), and 1 discontinuation of COC (discontinued during PK phase).

Serious AEs (SAEs) were reported in 8 patients and included ascites (n = 2; both grade 3), ileus, intestinal metastasis, myositis, nausea, and pneumonia (n = 1 each; all grade 3), and 1 patient with a grade 4 SAE of hypoglycemia. Two patients had SAEs considered to be related to the study drug (1 patient with a grade 3 SAE of ileus; 1 patient with a grade 3 SAE of myositis).

Four patients discontinued the study drug due to AEs (all were AEs related to the study drug). One patient discontinued the COC in the PK phase due to grade 3 AEs of ALT increased and AST increased. Three patients discontinued trametinib (all in the post‐PK phase), including 1 patient with grade 3 SAEs of myositis, 1 patient with a grade 3 SAE of fatigue, and 1 patient with a grade 2 SAE of decreased ejection fraction. Ten patients had at least 1 AE requiring dose adjustment and/or interruption. The most common (reported in ≥20% of patients) AEs leading to dose adjustment or interruption were gastrointestinal disorders (4 patients). With respect to AEs of special interest for trametinib, during the overall study, 12 (63.2%) patients had skin toxicity, 5 (26.3%) patients had hepatic disorders, 3 (15.8%) patients each had bleeding events and hypertension, respectively, and 1 (5.3%) patient had a cardiac‐related event. Of these, 5 patients experienced a grade 3 AE of special interest (1 hepatic failure, 1 increased ALT and AST, 1 increased blood bilirubin, 1 hypertension, and 1 increased blood pressure), 2 patients discontinued treatment due to an AE of special interest (1 decreased ejection fraction, 1 increased ALT and AST), and 2 patients interrupted treatment due to an AE of special interest (1 rash, 1 increased gamma‐glutamyl transferase). There were no clinically relevant changes in vital signs, ophthalmic examination, or ECG. Clinically significant abnormality in left ventricular ejection fraction based on echocardiogram and multigated acquisition scans was observed in 1 patient.

Overall, 8 deaths were reported in the study. Of these, 4 deaths occurred during treatment (1 during the PK phase; 3 during the post‐PK phase), and 4 occurred >30 days after the last study treatment. For 7 of the 8 deaths, the cause of death was the underlying cancer (including the death during the PK phase); the eighth death, occurring 171 days after the last study treatment, was due to an unknown reason.

## Discussion

This DDI study, which was conducted as part of a postapproval measure requested by the European Medicines Agency Committee for Medicinal Products for Human Use, assessed as primary objective the impact of trametinib on the exposure of NE and EE in women receiving COCs and trametinib. The study also assessed the potential impact of NE/EE‐containing COCs on the exposure of trametinib and its metabolite M5 as an exploratory objective, as well as the safety of trametinib in women with solid tumors who take COCs as a secondary objective.

Findings for the primary study objective show that steady‐state trametinib does not lead to a decrease in NE or EE exposure. Instead, repeat dosing of trametinib resulted in an increase in NE exposure of 20%, with no effect on EE exposure. The reason for the 20% increase in NE AUC is unknown. The 20% increase in NE exposure is not considered clinically relevant in terms of efficacy because a potential reduction, but not an increase, in COC exposure was the clinical concern in this study, as this might lead to contraception failure. Importantly, the 20% increase in NE exposure is also not considered clinically relevant in terms of safety, as NE doses higher than the one used in the present study (eg, up to 5 mg daily) are approved for the treatment of menstrual irregularities and endometriosis and have been shown to be well tolerated in long‐term studies for up to 5 years.^22^

The observed effect of repeat‐dose trametinib on the PK of COCs in this study in female individuals is in line with the expected in vivo effect of trametinib on COCs, which was based on preclinical in vitro data. Although trametinib has been shown to be a weak inducer of CYP3A4 with an EC_50_ of 1.7 μM in vitro (Novartis, unpublished data) the clinically relevant systemic concentration of trametinib is lower (≈0.04 μM^15^) than the observed in vitro induction value of CYP3A4, suggesting that an in vivo effect of trametinib on COC exposure is unlikely. Thus, the present study confirms that in patients receiving repeat dosing trametinib, exposure to NE or EE is not reduced, and therefore an impact on the contraceptive efficacy of a COC consisting of NE/EE is not expected.

Given that trametinib is largely administered as a combination therapy with the BRAF inhibitor dabrafenib, it may be relevant to establish the effect of the trametinib + dabrafenib combination on the PK of COCs. No data on the exposure of COC in patients receiving the combination of trametinib + dabrafenib are available. A clinical DDI study of dabrafenib and NE/EE‐containing oral contraceptives has not been performed, but in vitro, dabrafenib has been shown to be an inducer of CYP3A4, with an EC_50_ for CYP3A4 mRNA induction of 1.6 μM,^23^ which could render hormonal contraceptives ineffective. Therefore, female patients of reproductive potential are advised to use effective, nonhormonal contraception during dabrafenib treatment and for 2 to 4 weeks after the last dose.

Exploratory objectives of the present study were to assess the PK of trametinib and its metabolite M5 in the presence of COCs. Trametinib is metabolized by deacteylation and hydroxylation, most likely via CYP enzymes such as CYP3A,^17^ and it has been shown that steroids such as EE can act as inhibitors of CYP3A.^24^, ^25^, ^26^ In the present study, the geometric means of the trametinib PK parameters for AUC_tau_, AUC_last_, and C_max_ were 398 ng • h/mL, 346 ng • h/mL, and 27.4 ng/mL, respectively, with a median t_max_ of 2.01 hours. These values compare favorably with historic data of trametinib 2 mg when used in the absence of COC (geo‐mean AUC_0‐24_ 370 ng • h/mL; geo‐mean C_max_ 22.2 ng/mL; median t_max_ 1.75 hours).^15^ The metabolite M5 is unlikely to contribute to meaningful clinical activity of trametinib in women receiving concomitant COC because (1) M5 exposure was considerably lower relative to the parent (ratio of exposure of M5 to trametinib was 0.2), (2) the trametinib intrasubject variability was more than 30%, and (3) the trametinib exposure‐efficacy response relationship is relatively flat.^15^, ^17^

Finally, the present study also assessed the safety of trametinib in women with solid tumors taking COCs. The coadministration of trametinib and COC was generally well tolerated; safety signals observed in the present study were consistent with the known safety profile of trametinib and no new safety events were reported. The most common AEs suspected to be study drug related were skin and gastrointestinal disorders (rash, diarrhea, and nausea); SAEs suspected to be study drug related were rare (2/19 patients). The AEs reported in the study were mostly detectable by routine monitoring and manageable with supportive general care. Most deaths (7/8) occurring during the study were due to disease progression, and no treatment‐related deaths were reported.

## Conclusions

This DDI study in female patients with solid tumors shows that coadministration of trametinib with NE/EE‐based COCs results in a clinically nonrelevant 20% increase in NE exposure, with no change in EE exposure. The findings suggest that repeat dosing of trametinib does not impair the oral contraceptive efficacy of COCs, thus confirming previous in vitro observations that suggested no CYP3A4 induction activity of trametinib in vivo. The study also showed that coadministration of trametinib and COCs is generally well tolerated, with no new safety signals. In conclusion, the findings support the use of hormonal COCs in female patients with solid tumors who receive trametinib monotherapy.

## Conflicts of Interest

H.‐T.A. has been employed by HCA Healthcare UK/ Sarah Cannon Research Institute; and served as a speaker/advisor for Bayer, Beigene, Bicycle, Engitix, Guardant, iOnctura, Roche, and Servier.  D.T. has served as a consultant (honoraria) to Daiichi Sankyo, Lilly, Novartis, and Roche; and has received travel grants: AstraZeneca, Pfizer, and Roche.  C.L.‐P. has participated in a speakers’ bureau for Roche; and had travel and accommodation paid by Astellas Pharma, Angelini Pharma, and Bristol‐Myers Squibb. K.M. has participated in advisory boards/served as a consultant for Aravive, AstraZeneca/MedImmune, Eisai, Elevar, GSK/Tesaro, Genentech/Roche, Immunogen, Merck, Mersana, Myriad, Sorrento, OncXerna, and VBL Therapeutics; and received research funding from Lilly, Merck, and PTC Therapeutics.  J.d.V.‐G. has served as a consultant for AstraZeneca, MSD, Pierre Fabre, and Servier; and has received institutional research funding from Servier (all outside the submitted work).  X.X. was an employee of Novartis at the time the research was done and is currently an employee of Janssen. S.C., P.K.N., and P.I. are employees of Novartis.

## Funding

This study was sponsored by Novartis Pharmaceutical Inc.
